# Isotopic labeling-assisted metabolomics using LC–MS

**DOI:** 10.1007/s00216-012-6375-y

**Published:** 2012-09-26

**Authors:** C. Bueschl, R. Krska, B. Kluger, R. Schuhmacher

**Affiliations:** Center for Analytical Chemistry, Department for Agrobiotechnology (IFA-Tulln), University of Natural Resources and Life Sciences, Vienna, Konrad-Lorenz-Str 20, 3430 Tulln, Austria

**Keywords:** Bioanalytical methods, Mass spectrometry, Metabolomics, Liquid chromatography

## Abstract

Metabolomics has emerged as the latest of the so-called “omics” disciplines and has great potential to provide deeper understanding of fundamental biochemical processes at the biological system level. Among recent technological developments, LC–HRMS enables determination of hundreds to thousands of metabolites over a wide range of concentrations and has developed into one of the most powerful techniques in non-targeted metabolomics. The analysis of mixtures of in-vivo-stable isotopic-labeled samples or reference substances with un-labeled samples leads to specific LC–MS data patterns which can be systematically exploited in practically all data-processing steps. This includes recognition of true metabolite-derived analytical features in highly complex LC–MS data and characterization of the global biochemical composition of biological samples. In addition, stable-isotopic labeling can be used for more accurate quantification (via internal standardization) and identification of compounds in different organisms.

## Metabolomics: a brief introduction

The objective of metabolomics is comprehensive, qualitative and quantitative analysis of all the low-molecular-weight metabolites of a living cell, organ, or whole organism [1]. The term “metabolome” has been defined by analogy with the genome and refers to the complete set of metabolites of a biological system [2, 3]. Thus metabolomics can be regarded as characterization of the metabolome. Although genomes have been sequenced for many organisms, it is currently not possible to measure the whole metabolome of a biological system at once, because of analytical–methodical limitations and the highly diverse nature of the metabolites.

In this respect, two different metabolomics concepts can be distinguished: targeted and non-targeted. In targeted approaches, abundances of metabolites of a set of predefined known substances are determined. Such an approach enables absolute quantification but is usually limited to metabolites which are available as authentic reference standards. In contrast, non-targeted approaches try to find mass spectrometric features of all detectable compounds, including those unknown at the time of sample measurement. This approach therefore has the advantage of probing the entire metabolic space and can obtain relative abundances of several hundreds to thousands of known and unknown metabolites [4].

Currently, most non-targeted metabolomics studies use liquid chromatography coupled to high-resolution mass spectrometry (LC–HRMS). This combination enables the detection of the highest number of metabolites and requires only small amounts of the biological sample [5]. The combination of electrospray ionization (ESI) with full-scan LC–MS at unit resolution results in a large number of solvent-related cluster ions and other non-metabolite-related signals which might interfere with masses of true metabolite ions. For this reason, low resolution ESI LC–MS is usually restricted to targeted approaches in which MS–MS modes are mostly applied. Most of the applications described in this article were, however, used for non-targeted analysis of biological samples using full-scan LC–HRMS.

Sample measurements with modern analytical instrumentation result in huge amounts of data, which can no longer be evaluated manually. Data-processing steps serve to reduce data complexity and include numerous elements, for example feature extraction, spectrum deconvolution (i.e., grouping of ions which originate from the same metabolite), retention time (*R*_t_) alignment of chromatographic peaks across different runs, and internal standardization for quantification. The final result of data processing is a data matrix, a table that contains all samples and analytical features (i.e., metabolite-derived signals). The ultimate objective of a metabolomics study is to detect as many metabolites as possible and to link the differently expressed metabolites to a variety of experimental factors. Therefore, reliable annotation and/or identification of the detected metabolites is essential.

Despite the availability of several software tools for data processing [6–11] and improved measurement techniques, several limitations remain.

### From the extraction of metabolite-derived analytical features to the annotation and identification of metabolites

A first step of data processing in non-targeted metabolomics approaches is to extract as many features as possible. The term “feature” denotes a two dimensional bounded signal consisting of a chromatographic peak (i.e., retention time) and an MS peak (i.e., *m*/*z* value) [12]. The comprehensive and reliable extraction of metabolite-derived features of true biological origin however, remains a very difficult task. A major limitation is the large amount of non-metabolite-related noise and background signals. It has been estimated that in LC–electrospray ionization–MS (LC–ESI–MS), as little as 10 % of the signals are of true biological origin [13]. Consequently, most features are not associated with true metabolites but can hardly be recognized as such, because their analytical characteristics are the same as for features of true biological origin. Furthermore, a single metabolite leads to more than one ion species—e.g., isotopic peaks, adducts, in-source fragments. Therefore, data processing requires the assignment of metabolite ions and the grouping of features into deconvoluted spectra before meaningful annotation of metabolites can be achieved. Typically, feature abundances are estimated by peak integration and the resulting peak areas are used for relative quantification and comparison of different experimental states.

After feature extraction and spectral deconvolution, annotation and/or identification of metabolites is essential, but is one of the most challenging tasks of any metabolomics experiment [14–16]. Metabolite annotation usually uses a search for one or more molecular properties (e.g., accurate mass, sum formula) against comprehensive databases (DB), whereas identification also requires confirmation by measurement of an authentic standard under identical analytical conditions and/or comparison of MS–MS fragmentation patterns.

DB search approaches seldom provide unique identifications, since usually more than one substance exists for a particular mass or sum formula, because of the great number of combinations of one sum formula into numerous structural formulas. Furthermore, because non-targeted metabolomics experiments yield both known and unknown metabolites, it can be expected that a significant number of metabolites is missing from the DBs and thus remains uncharacterized [5]. Additional MS–MS measurements are required to provide structure information. Unfortunately, MS–MS measurements are very labor-intensive tasks even for only a small subset of putative metabolites and the resulting MS–MS spectra are difficult to interpret. Furthermore, for LC–MS very few databases with authentic MS–MS spectra are available, again reducing the number of identifiable metabolites.

With regard to quantification of metabolites by LC–ESI–MS, matrix effects, or signal suppression or enhancement (SSE) (for recent review see, e.g., Refs. [17] and [18]), limit the accuracy and reliability of quantitative measurements within and between different measurement sequences. SSE is caused by the presence of (endogenous or exogenous) co-eluting components in the ion source of the mass spectrometer and has been attributed to numerous mechanisms including competition for “charges” between analytes and interfering compounds or a change of viscosity and/or surface tension of the droplets in the ion source [17, 18].

### The potential of stable isotopic labeling to meet current challenges in LC–MS-based metabolomics research

In view of these limitations, there is a strong need both for innovative approaches to the analytical measurement of biological samples and for the development of novel, improved data-processing algorithms and their implementation in the form of user-friendly software tools, especially for non-targeted metabolomics.

**Fig. 1:**
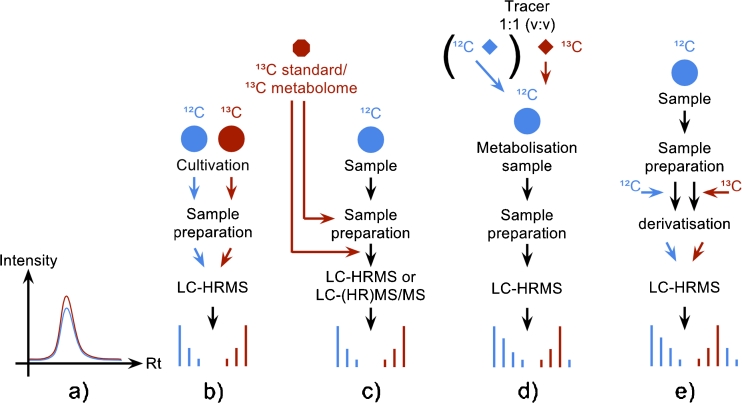
Different strategies for using SIL to assist metabolomics studies. **a** ^13^C, ^15^N, and ^34^S-enriched substances are not chromatographically separated from the corresponding natural isotopologues, thus the non-labeled and the labeled isotopologues elute at the same retention time with identical peak profiles. **b** For non-targeted annotation of an organism’s metabolome the organism can be cultivated in parallel using differently isotopologue-enriched nutrition sources (e.g., ^12^C and ^13^C glucose as sole carbon source). The extracts are subsequently mixed and measured with LC–HRMS. The resulting data pattern helps in the extraction of true biological signals. **c** Absolute compound quantification using an authentic, labeled standard or relative quantification using a stock of globally labeled sample extract of the same organism for inter-experiment comparison. **d** Metabolism experiment using natural and fully labeled tracer substances enables metabolism studies and greatly helps to separate products of metabolism from other biological signals. In contrast with metabolism studies, fluxomics experiments only spike with the labeled tracer. **e** Derivatisation using non-labeled and labeled derivatisation agents enables rapid recovery of many metabolites belonging to the same chemical groups (e.g., alcohols, acids …)

**Fig. 2:**
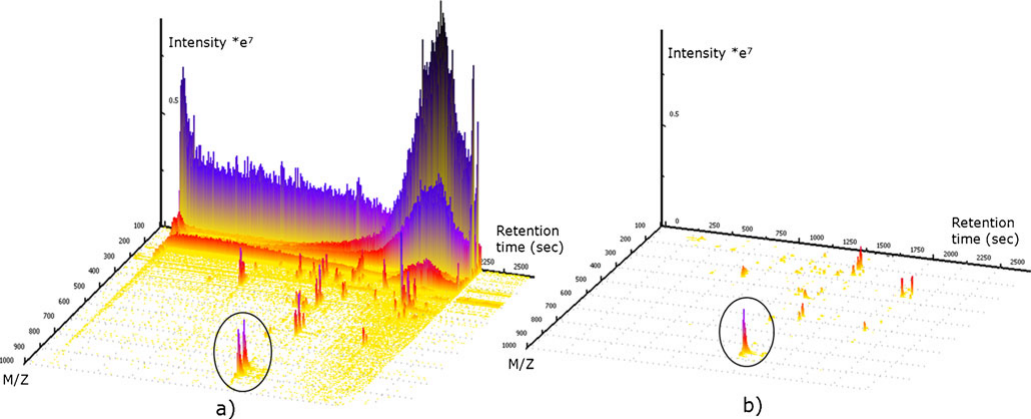
3D Plots obtained from *F. graminearum*. **a** Unprocessed LC–HRMS full-scan chromatograms of a mixture of supernatants from cultivation of *F. graminearum* on both ^12^C and ^13^C glucose. The *circle* marks an ion pair (the ^12^C and the corresponding ^13^C-labeled monoisotopic ions) originating from the same metabolite. After processing the spectrum with MetExtract, only ions having a labeled pendant are not removed (**b**). Thus, only the non-labeled ions remain in the circled area. (3D view generated with Ref. [37])

In this respect, stable isotopic labeling (SIL) is a very promising and increasingly popular technique in metabolomics research, which is perfectly suited to be combined with GC–MS and LC–MS. Stable isotope techniques were preceded by the use of radioactive isotopes in biochemical research. The development of robust and sensitive GC–MS and LC–MS instrumentation together with easier and safer handling procedures (i.e., organizational restrictions, human health concerns), quickly increased the use of radio isotopes [19]. First applications of SIL in proteomics and protein labeling demonstrated the huge advantages of this technique [20] and researchers therefore adopted SIL in a variety of metabolomics techniques. In this respect ^13^C is the most commonly used isotope for SIL-assisted experiments, because ^13^C-labeled isotopologues cannot be chromatographically separated from their natural analogues (Fig. 1a). Moreover, carbon is a constituent of every metabolite and its transfer between biological entities follows well known rules [19]. Although ^15^N and ^34^S are also well suited to investigation of all nitrogen or sulfur-containing compounds and their bio-transformation by living systems, ^2^H and ^18^O are less frequently used for labeling of metabolites in metabolomics, because they frequently tend to be exchanged by hydrogen and oxygen from surrounding water. Moreover deuterated metabolites do not always perfectly co-elute with their non-labeled analogues [19], rendering the extent of labeling and the results obtained from subsequent data processing difficult to interpret. Figure 2 shows a three dimensional view of an LC–HRMS chromatogram of the supernatant from the agricultural important fungus *F. graminearum*, which was cultivated in parallel on ^12^C and ^13^C glucose medium. Identical aliquots of the ^12^C and ^13^C supernatant were mixed and subsequently measured using an LTQ Orbitrap XL. As one can see in the circled area, the LC elution pattern for the ^12^C (natural) and ^13^C-labeled metabolite ions of the same metabolite are identical but have a defined *m*/*z* shift (861.3835 for the ^12^C ion and 897.5043 for the corresponding ^13^C ion). Because ^12^C and ^13^C-labeled glucose were used for cultivation, the number of carbon atoms in this particular ion can be calculated from the *m*/*z* difference between the two monoisotopic mass peaks. For this example, the *m*/*z* difference of 36.1208 suggests 36 carbon atoms for this putative metabolite.

To further confirm that the two isotopologue ions belong to the same putative metabolite, their carbon-isotopic distributions are of interest. Whereas descending isotopic distributions towards higher *m*/*z* values (the abundance of natural occurring ^12^C is approximately 98.93 % and thus the abundance of ^13^C is 1.07 %) are observed for all natural ^12^C substances, ^13^C-labeled substances do not have descending isotopic distributions, because their most abundant carbon isotope is ^13^C and only the minority of carbon atoms are ^12^C isotopes (the abundance of ^13^C and ^12^C is highly dependent on the enrichment method and cannot be generalized in the same way as natural carbon abundances). Thus ^13^C-labeled monoisotopic ions are not only heavier but also have ascending isotopic distribution toward higher *m*/*z* values, because ^13^C isotopes are replaced by the lighter ^12^C isotopes. These two mirror symmetric isotopic distributions, which are only present for SIL using carbon, confirm that both ions are successors of the same metabolite and enable the number of carbon atoms to be easily determined. Depending on the MS instrument used, the relative abundance of the isotopic distributions are more or less accurately recorded. In general, TOF instruments yield more accurate relative abundances of isotopic peaks than Orbitrap or other FT mass analyzers, whereas the resolving power and thus mass accuracy are higher with FT-MS instruments. The theoretical isotopic-abundance pattern for a specific isotopologue in comparison with the substance’s most intense isotopic peak is calculated by use of the formula:

$$ P\left( {\overrightarrow a, \overrightarrow s, \overrightarrow p, \overrightarrow {{p_e}} } \right) = \mathop{\varPi}\limits_{{i = 0}}^{{\left| a \right|}} \frac{{{p_{{ei}}}^{{{a_i} - {s_i}}}{p_i}^{{{S_i}}}\left( {\matrix{ {{a_i}} \\ {{s_i}} \\ }<!end array> } \right)}}{{{p_{{ei}}}^{{{a_i}}}}} $$

where *a*_*i*_ is the number of atoms of the *i*th element in the substance, *s*_*i*_ is the number of substitutions of the most abundant isotopologue with less abundant isotopes of the *i*th element, *p*_*i*_ is the isotopic purity of the used stable isotope of the *i*th element, and *p*_*ei*_ is the isotopic purity of the most abundant isotope of the *i*th element (e.g., the relative abundance of the first isotopologue (^13^C_1_) compared with its ^12^C monoisotopic peak for an ion having 24 carbon atoms is P([24], [1], [0.0107], [0.9893]) = 25.68 %. The same isotopologue having additionally replaced one of nine oxygen atoms by ^17^O (natural abundances for ^16^O and ^17^O are 99.962 % and 0.038 %) is P([9, 24], [1, 1], [0.0107, 0.00038], [0.9893, 0.99962]) = 0.08 %). However, very low isotopic abundances (e.g., ^17^O, ^36^S, ^2^H …) may be of theoretical interest but, in general, do not have to be considered practically in LC–HRMS analysis because they are not observable with most current MS instrumentation.

Furthermore, the mirror symmetric isotopic distributions in the case of carbon SIL are very helpful if the substances of interest contain heteroatoms such as sulfur, nitrogen, or calcium. Without the SIL process, heteroatom isotopologues cannot usually be separated from the more intense ^13^C isotopologues of the natural, ^12^C ions even at a high MS resolving power of 100,000. With carbon SIL additional isotopic mass peaks originating from heteroatom isotopes (e.g., ^15^N, ^34^S) can be observed next to the ^13^C ion at higher *m*/*z* values. This benefit is very interesting for non-targeted metabolomics because it provides further information about the elemental composition of the detected ion species.

Apart from non-targeted metabolomics, feature extraction and annotation, SIL is also perfectly suited to absolute quantification. Because the natural and spiked labeled reference standard perfectly co-elute, the ratio of their peak areas is used for absolute quantification. Furthermore, because the standard is added to the biological sample, its concentration is known. Using the ratio of the two isotopologue peak areas and the amount of reference standard, absolute quantification of the natural isotopologue in the sample can be achieved.

### Examples of applications of SIL

All of the following SIL-assisted approaches involve analysis of mixtures of labeled and non-labeled samples and are based on enrichment of the respective mass spectra with the isotopic pattern of the SIL analogue(s). Depending on the purpose of the labeling experiment (Fig. 1b–e), the degree of isotopic enrichment, metabolic fate of the tracer(s), and other factors, the resulting mass spectra might be affected in different ways but all have the SIL-specific data pattern exemplified in Fig. 1. The mass spectra might just contain (slightly) altered relative intensities of individual isotopologues or pairs of clearly separated isotopic patterns of the natural compounds and their corresponding fully labeled analogues.

In the following four main areas involving SIL and LC–MS in metabolomics research will be presented with the focus on LC–HRMS.

#### SIL-assisted whole metabolome studies (Fig. 1b)

For reliable extraction and annotation of putative metabolites in a biological sample, non-labeled and labeled cultures of the organism of interest are mixed and measured by use of LC–HRMS in the full-scan mode. Whole labeled metabolomes of specific organisms can be obtained when a fully labeled substrate, for example ^13^C_6_ glucose forms the sole carbon source of, e.g., bacteria or filamentous fungi. Such in vivo labeling of microbes has been successfully used to accomplish internal standardization for quantification, to circumvent problems of signal suppression in ESI–MS [21]. For this purpose, in-vivo-labeled samples can either be cultured in parallel to every experimental sample and condition and subsequently mixed with non-labeled analogues, or prepared by producing a large quantity of labeled culture with which non-labeled experimental samples can be spiked [22, 23].

Recently, in-vivo labeling has also been extended to plants and used to facilitate the assignment of the elemental composition of metabolite ions by accurate mass measurements in combination with database search [24, 25]. Moreover, as we have demonstrated in our own recent work, in-vivo labeling of fungal culture samples provides a powerful approach for the automated global extraction of all metabolite-derived MS signals from LC–HRMS raw data by discrimination of true metabolite-related from non-specific analytical features [26].

#### SIL improves absolute quantification (Fig. 1c)

In targeted analysis, SIL reference standards can be used for internal standardization of peak intensities in trace analysis by use of LC–HRMS or LC–(HR)MS–MS [27, 28], making this the most common use of SIL. Use of authentic, labeled reference standards enables easy, rapid, and absolute quantification of the respective metabolites even in complex matrixes.

#### In vivo metabolism studies (Fig. 1d)

In non-targeted metabolomics, uniformly labeled substances in combination with their natural pendants are added to biological samples during cultivation and used as tracers to study their metabolic fate [4]. This technique has been used in targeted approaches to study metabolic pathways and fluxes of the central metabolism [29, 30]. Moreover, labeled tracers have also been used to study the bio-transformation of secondary metabolites [31] and xenobiotics [32]. Without use of SIL for this kind of research, searching for and detecting these products of metabolism in complex full-scan LC–HRMS data by non-targeted approaches would be almost impossible. Without the use of SIL one would have to subtract chromatograms without the spiked compound from those obtained after measurement of samples with the specific compounds added. Although possible in principle, the latter approach results in numerous false-positive findings because of fluctuations in MS signals from measurement to measurement.

#### SIL-assisted derivatisation (Fig. 1e)

Another recently introduced SIL-based technique in combination with LC–HRMS is the use of labeled reagents for derivatisation which enables non-targeted screening for all compounds belonging to a specific chemical group (e.g., alcohols, acids …). The general workflow for a SIL derivatisation step is to split the biological sample into two identical aliquots and perform derivatisation separately with labeled and non-labeled derivatisation reagents, and mix and measure them jointly. Thus, one not only gains all the benefits derivatisation has but also the benefits of SIL which, in this case, are improved metabolite feature extraction in the highly complex LC–HRMS data [33, 34] and an estimate of the exchanged functional groups.

Despite the high potential of labeling-assisted metabolomics approaches, only a few data processing tools have been published which specifically exploit the labeling-associated data pattern. Moreover, these tools are limited to targeted metabolomics and fluxomics approaches. For targeted GC–MS-based metabolomics of cell cultures, Hiller and colleagues published a method for study of the fate of labeled tracer compounds through central metabolism [35]. For targeted LC–MS studies, commercial software (IROA) exists which has been designed to use mixtures of in-vivo labeled and non-labeled biological culture samples to assign differently expressed metabolites in differently treated biological samples. The software calculates and graphically illustrates the intensity ratios of the principal ions from predefined isotopologue signal pairs [36]. Another software product also utilizing ^13^C labeling is MetMax [11], which has been used to analyze the dynamics of CO_2_ uptake by *Chlamydomonas reinhardtii* using GC × GC–TOF-MS. To the best of our knowledge, the recently developed MetExtract software is, to date, the only publicly available tool for non-targeted, automated global detection of metabolite-derived LC–HRMS signals originating from natural and stable isotopically labeled analogues and their assignment to true biological metabolites [26].

Although LC–MS experiments using stable isotopically labeled compounds in combination with their naturally occurring pendants is already a well established and frequently used technique, the most limiting disadvantage is the relatively high cost associated with enrichment of compounds with some stable isotopes. Furthermore, a stable isotopic labeled source which can be used for in-vivo labeling experiments or as an internal standard may not be available commercially. Authentic labeled reference standards or nutrition sources have to be synthesized or harvested from organisms and subsequently purified, which again is a cost-intensive exercise. For reference standards, the second major limitation is availability because only a very small subset of all needed reference standards are available, which limits their advantage. Another limitation of SIL and whole-metabolome experiments is the requirement to cultivate the organisms in parallel—once with the natural and once with the stable isotope enriched nutrition source—to be able to recover experiment or condition-specific metabolites of the organism of interest.

## Outlook

Although SIL-based analytical approaches have been developed and frequently used in both proteomics and biochemical research for many years, the metabolomics community started to fully exploit the potential of this technique only recently. Considering the numerous benefits and advantages of SIL, we expect its increased application, particularly for non-targeted metabolomics research. Although stable isotopically labeled compounds or biological samples are quite expensive, they offer many benefits for analytical chemists working with LC–MS. The unique and undistinguishable data pattern obtained from natural and fully labeled isotopologues of a compound drastically simplifies data processing. Furthermore, in target analysis the addition of labeled authentic standards enables absolute quantification of substances in biological samples. For non-targeted metabolomics SIL-based LC–HRMS approaches enable global data extraction and feature annotation for true metabolites and can help to improve precision in relative quantification. With further improved analytical instrumentation and customized data-processing software for SIL-derived data patterns, this technique has the potential to be of crucial importance in the new discipline of metabolomics.
